# Comparative Study on the Protective Effect of Thiamine and Thiamine Pyrophosphate Against Hydroxychloroquine-Induced Cardiomyopathy in Rats

**DOI:** 10.3390/life16010037

**Published:** 2025-12-25

**Authors:** Izzet Emir, Bulent Yavuzer, Bahadir Suleyman, Cengiz Sarigul, Ali Sefa Mendil, Esra Tuba Sezgin, Durdu Altuner, Cebrail Gursul, Halis Suleyman

**Affiliations:** 1 Department of Cardiovascular Surgery, Faculty of Medicine, Erzincan Binali Yıldırım University, Erzincan 24100, Turkey; izzet.emir@erzincan.edu.tr; 2 Department of Pharmacology, Faculty of Medicine, Erzincan Binali Yıldırım University, Erzincan 24100, Turkey; bulent.yavuzer@erzincan.edu.tr (B.Y.); bsuleyman@erzincan.edu.tr (B.S.); daltuner@erzincan.edu.tr (D.A.); 3 Department of Medical Biochemistry, Faculty of Medicine, Erzincan Binali Yıldırım University, Erzincan 24100, Turkey; cengiz.sarigul@erzincan.edu.tr; 4 Department of Pathology, Faculty of Veterinary Medicine, Erciyes University, Kayseri 38039, Turkey; 4022140002@erciyes.edu.tr; 5 Anesthesia Program, Vocational School of Health Services, Erzincan Binali Yıldırım University, Erzincan 24036, Turkey; esra.demir@erzincan.edu.tr; 6 Department of Physiology, Faculty of Medicine, Erzincan Binali Yıldırım University, Erzincan 24100, Turkey; cgursul@erzincan.edu.tr

**Keywords:** cardiotoxicity, hydroxychloroquine, lactate dehydrogenase, oxidative stress, rats, Wistar, thiamine, thiamine pyrophosphate, troponin I

## Abstract

**Background:** Hydroxychloroquine (HCQ), widely used in autoimmune and inflammatory diseases, has been associated with cardiotoxicity driven by oxidative, mitochondrial, and metabolic disturbances. However, no comparative evidence exists regarding whether thiamine, thiamine pyrophosphate (TPP), or their combination (TTPC) can mitigate HCQ-induced myocardial injury. **Objective:** This study examined the biochemical and histopathological effects of thiamine, TPP, and their combination in a rat model of HCQ-induced cardiomyopathy. **Methods:** Thirty male Wistar rats were assigned to five groups: healthy control, HCQ, thiamine + HCQ, TPP + HCQ, and TTPC + HCQ. Thiamine (20 mg/kg, intraperitoneal), TPP (20 mg/kg, intraperitoneal), or TTPC (20 mg/kg each, intraperitoneal) was administered once daily, followed by HCQ (120 mg/kg, oral, twice daily). After seven days, cardiac tissue was analyzed for MDA, tGSH, SOD, and CAT, while serum TnI, lactate, and LDH were measured from tail-vein blood samples. Cardiac samples underwent histopathological examination. **Results:** HCQ exposure markedly increased MDA, TnI, LDH, and lactate levels while reducing tGSH, SOD, and CAT, indicating severe oxidative and metabolic insult. Thiamine co-treatment failed to ameliorate these disturbances. Conversely, TPP restored redox balance, attenuated biomarker elevations, and improved cardiac biochemical profiles. TTPC produced comparable improvements but did not exceed those of TPP alone. Histopathologically, HCQ caused pronounced myocyte degeneration and mononuclear infiltration, whereas TPP and TTPC groups showed only mild inflammatory changes with preserved myocardial architecture. **Conclusions:** HCQ induces a marked redox imbalance accompanied by well-defined histopathological myocardial degeneration. TPP afforded robust cardio-protection, whereas thiamine offered no meaningful benefit. Collectively, these findings position TPP as a biologically plausible, clinically relevant candidate for mitigating HCQ-induced cardiomyopathy.

## 1. Introduction

Hydroxychloroquine (HCQ) is a drug utilized in the clinical management of malaria and a broad range of autoimmune diseases, such as rheumatoid arthritis, systemic lupus erythematosus, antiphospholipid syndrome, and primary Sjögren’s syndrome, in addition to various inflammatory diseases [1]. HCQ enters the cell predominantly in its protonated form, and its intracellular concentration exhibits an inverse relationship with the surrounding pH. Consequently, it accumulates within acidic intracellular organelles, including endosomes, lysosomes, and Golgi-derived vesicles, thereby increasing their luminal pH [2]. The precise mechanism of action of HCQ has not yet been fully elucidated. However, HCQ is known to block Toll-like receptors in dendritic cells, elevate intracellular pH, inhibit the excessive activation of B and T lymphocytes, and reduce the production of proinflammatory cytokines [2,3]. Although HCQ is widely regarded as safe, accumulating evidence suggests that its use may be associated with adverse effects affecting ophthalmic, dermatological, gastrointestinal, neurological, and cardiac organ systems [4]. Cardiotoxic effects associated with the prolonged and high-dose use of HCQ include bradycardia, tachycardia, QT interval prolongation, torsade de pointes, atrioventricular block, and cardiomyopathy [5]. Cardiomyopathy is one of the undesirable adverse effects that may necessitate the discontinuation of HCQ therapy [6]. According to Akarsu, prolonged exposure to HCQ, particularly at high cumulative doses, may give rise to cardiac adverse effects that at times necessitate the discontinuation of the drug; however, such cardiotoxic manifestations are not invariably reversible [7]. The literature further emphasizes that cases of HCQ-induced cardiomyopathy have been documented, underscoring the need for the careful monitoring of cardiac function in patients receiving long-term therapy [8]. A previous study demonstrated that HCQ induces muscle and nerve injury, an effect attributed to its ability to elevate oxidative stress parameters [9]. The literature indicates that HCQ can impair the function of organelles, such as lysosomes, and trigger mitochondrial apoptotic signaling, ultimately resulting in cardiomyocyte dysfunction [10,11]. Another alteration observed in cardiomyopathies involves changes in lactate dehydrogenase (LDH) enzyme activity. As is well known, LDH catalyzes the forward conversion of pyruvate to lactate and the reverse conversion of lactate to pyruvate [12]. A case of cardiomyopathy has been reported in a patient with mitochondrial encephalomyopathy, lactic acidosis, and stroke syndrome, showing elevated myocardial oxidative stress [13]. For this reason, investigations into the role of lactate in cardiovascular disease have gained substantial scientific momentum [14]. In living organisms, the formation of lactate occurs through the conversion of intracellular alanine and glucose into pyruvate via the action of the LDH enzyme [15,16]. Previous studies have associated impaired cardiac function with alterations in LDH activity [17]. Zhang et al. reported that elderly patients presenting with elevated baseline serum LDH levels exhibited a higher risk of being diagnosed with heart failure during follow-up [18]. Biguetti et al. reported elevated LDH enzyme activity in cases of HCQ-associated myopathy [19].

In our study, the agent we will investigate for its potential protective effects against HCQ-induced cardiotoxicity in animals is thiamine, which is known as vitamin B1 [20]. Thiamine is converted in the body into its active form, thiamine pyrophosphate (TPP), by the enzyme thiamine pyrophosphokinase [21]. As is well known, TPP serves as a cofactor for pyruvate dehydrogenase and participates in the conversion of pyruvate to acetyl–coenzyme A. In the absence of TPP, pyruvate is converted to lactate, and the resulting accumulation of lactic acid may lead to the development of lactic acidosis [22]. Collectively, the evidence available in the literature indicates that thiamine and TPP could hold therapeutic potential in managing HCQ-induced cardiomyopathy. Moreover, a review of the existing literature reveals no studies specifically investigating the effects of thiamine or TPP in the treatment of HCQ-induced cardiomyopathies. Therefore, this study aims to investigate and comparatively evaluate the potential effects of thiamine, TPP, and their combination (TTPC) on the development of HCQ-induced cardiomyopathy in rats.

## 2. Materials and Methods

### 2.1. Animals

A total of thirty male Wistar albino rats, confirmed to be 9–10 weeks of age and weighing 255–272 g at baseline, were assigned to this study in a controlled manner in order to ensure a uniform experimental cohort and reduce potential confounding related to age- or weight-associated physiological variability. All animals were obtained from the Experimental Animals Application and Research Center of Erzincan Binali Yıldırım University (Erzincan, Turkey). Rats were randomly allocated to five experimental groups (*n* = 6 each), with efforts taken to maintain comparable baseline body weight distributions among groups to minimize confounding variability. Prior to the onset of experimental interventions, the animals were allowed a one-week acclimatization period and were kept in standard wire-mesh cages (20 cm high × 35 cm wide × 55 cm long; 1925 cm^2^ floor area), with six rats accommodated in each enclosure. Environmental conditions were strictly controlled, with a 12 h light/12 h dark photoperiod, an ambient temperature of 22 ± 2 °C, and relative humidity maintained within the 30–70% range. Animals were provided ad libitum access to standard laboratory chow (Bayramoglu Feed and Flour Industry Inc., Erzurum, Turkey) and tap water throughout this study. All experimental interventions were carried out in the accredited laboratory units of the Experimental Animal Application and Research Center of Erzincan Binali Yıldırım University. The study protocol was designed and executed in full compliance with Directive 2010/63/EU of the European Parliament, which governs the protection of animals used for scientific purposes (Protocol ID: 2016-24-199). All methodological and reporting practices adhered to the ARRIVE (Animal Research: Reporting of In Vivo Experiments) guidelines [23].

### 2.2. Reagents and Chemicals

All reagents and chemicals used in this study were of analytical grade and were obtained from certified suppliers. Thiopental sodium (Pental Sodyum^®^, 0.5 g vial) was purchased from Menarini Health and Pharmaceuticals Industry Trade Inc. (Istanbul, Turkey). Hydroxychloroquine sulfate (Plaquenil^®^, 200 mg tablet) was supplied by Sanofi Pharmaceuticals Industry and Trade Inc. (Istanbul, Turkey). Thiamine (Thiamine chloride^®^, 50 mg/mL injectable solution) and thiamine pyrophosphate (Cocarboxylase hydrochloride^®^, 50 mg/2 mL injectable solution) were sourced from BioPharma (Kyiv, Ukraine).

### 2.3. Experimental Design and Randomization

The number of animals included in this study was established in accordance with the principle of using the minimum number of subjects necessary to obtain reliable and reproducible outcomes, in full alignment with the 4R framework (Reduction, Refinement, Replacement, and Responsibility) [24]. Exclusion criteria were delineated separately for each of the two phases of the experimental protocol. In the pre-experimental phase, animals exhibiting abnormal posture, reduced spontaneous activity, or injuries resulting from aggressive cage-mate interactions were excluded prior to randomization and the initiation of treatment. Peri- and post-experimental exclusion criteria covered a range of events that could compromise data integrity or animal welfare. These included unexpected mortality or anesthesia- or drug-related complications occurring before the scheduled endpoints; procedural errors during dosing, such as unsuccessful oral gavage or injection-related extravasation; deviations from the designated treatment regimen or incomplete delivery of study compounds; excessive weight loss exceeding 15–20% of baseline, dehydration, or clinical indicators of systemic illness; severe distress behaviors, including self-mutilation or persistent vocalization suggestive of uncontrolled pain; failure to complete behavioral assessments due to non-compliance or motor deficits unrelated to experimental interventions; and loss of tissue integrity during sample collection or processing that would impede reliable histological or biochemical analysis. These criteria were consistently enforced throughout the intervention phase and during subsequent data assessment. None of the animals met the predefined exclusion criteria during either the pre-experimental or the peri- and post-experimental phases; therefore, no subjects were removed from this study. Randomization was carried out using a random number table to guarantee an unbiased distribution of animals across groups. To further reduce potential confounding factors and systematic bias, each cage and individual animal was assigned a numerical code that was preserved consistently throughout the entire experimental period.

### 2.4. Experimental Groups

The experimental design consisted of five defined groups: C (healthy control); HCQG (120 mg/kg HCQ, oral); TH + HCQ (20 mg/kg thiamine, intraperitoneal + 120 mg/kg HCQ, oral); TP + HCQ (20 mg/kg TPP, intraperitoneal + 120 mg/kg HCQ, oral); and TH + TP + HCQ (20 mg/kg thiamine, intraperitoneal + 20 mg/kg TPP, intraperitoneal + 120 mg/kg HCQ, oral).

### 2.5. Experimental Procedure

The animals in the TH + HCQ (*n* = 6) and TP + HCQ (*n* = 6) groups received intraperitoneal injections of thiamine and TPP at doses of 20 mg/kg, respectively. The TH + TP + HCQ group (*n* = 6) received an intraperitoneal combination of thiamine and TPP (TTPC), each administered at a dose of 20 mg/kg. The dose and administration route of thiamine and TPP were based on validated experimental models demonstrating their consistent antioxidant, metabolic, and tissue-protective efficacy [25]. Animals in the C (*n* = 6) and HCQG (*n* = 6) groups received distilled water as a vehicle, administered via the same route and in the same volume. One hour after the administration of thiamine, TPP, TTPC, or distilled water, animals in the TH + HCQ, TP + HCQ, and TH + TP + HCQ groups received HCQ at a dose of 120 mg/kg, which was administered orally via gastric gavage twice daily. Thiamine, TPP, and TTPC were administered once daily. This procedure was carried out daily for a total duration of 7 days. The present dosing regimen was selected based on previously published studies demonstrating that the administration of HCQ at 120 mg/kg twice daily [26] for 7 consecutive days [27] reliably induces tissue oxidative stress in experimental rat models. Moreover, the reported oral LD_50_ of HCQ in rats is 1240 mg/kg, indicating a substantial safety margin relative to the dose employed in the present study [28]. Upon completion of the treatment period, all animals were euthanized under deep anesthesia induced by thiopental sodium at a dose of 50 mg/kg, and cardiac tissues were immediately harvested. The levels of malondialdehyde (MDA), total glutathione (tGSH), superoxide dismutase (SOD), and catalase (CAT) were determined in the excised cardiac tissues. In addition, the harvested tissues underwent histopathological assessment. The data generated from each experimental group were subjected to intergroup comparison to allow comprehensive evaluations of this study’s outcomes.

### 2.6. Biochemical Analyses

#### 2.6.1. Preparation of Samples

Cardiac tissue was carefully excised from each animal and briefly rinsed with ice-cold 0.9% sodium chloride solution to remove residual blood and tissue debris. After weighing approximately 100 mg of tissue collected from each rat, the samples were minced into small pieces, snap-frozen in liquid nitrogen, and pulverized into a fine powder using a pre-cooled mortar and pestle. Following pulverization, the tissue powder was homogenized in phosphate-buffered saline (PBS, pH 7.4) prepared at a 1:10 (*w*/*v*) ratio to ensure consistent extraction efficiency. The homogenates were briefly vortexed (10 s) and centrifuged at 10,000× *g* for 20 min at 4 °C to obtain the supernatant. The obtained supernatant was carefully collected and stored at −80 °C until biochemical analyses were conducted. To maintain analytical consistency and facilitate comparisons across groups, all biochemical parameters were normalized to total protein levels, with MDA and tGSH reported as nmol/mg protein and SOD and CAT as U/mg protein.

Troponin I (TnI), lactate, and lactate dehydrogenase (LDH) levels were determined in blood samples collected from the tail veins.

#### 2.6.2. Determination of MDA, tGSH, SOD, CAT, and Total Protein Levels in Cardiac Tissue

MDA and tGSH levels and SOD enzymatic activity in cardiac tissue were quantified using commercially available, rat-specific ELISA kits (Cayman Chemical Co., Ann Arbor, MI, USA; catalog numbers: 10009055 for MDA, 703002 for tGSH, and 706002 for SOD), in full accordance with the manufacturer’s protocols. Catalase enzymatic activity was assessed according to the method described by Goth [29]. Total protein levels were quantified via the Bradford method [30] based on the interaction of Coomassie Brilliant Blue G-250 dye with protein molecules. Absorbance was recorded spectrophotometrically at 595 nm, and the calculated protein concentrations served for normalization of all biochemical parameters.

#### 2.6.3. Determination of Serum TnI Levels

Serum TnI levels were quantified using the VIDAS^®^ TnI Ultra assay kit (bioMérieux, Marcy-l’Étoile, France) based on the Enzyme-Linked Fluorescent Assay (ELFA) principle. The method utilizes a Solid Phase Receptacle (SPR) coated with monoclonal anti-TnI antibodies, enabling selective immunocapture of TnI from serum samples. All analytical stages—including sample aspiration, incubation, immunocomplex formation, automated washing steps, the addition of the alkaline phosphatase-labeled detection antibody, and enzymatic fluorescence development—were performed automatically via the VIDAS^®^ analyzer (bioMérieux, Marcy-l’Étoile, France) using the ready-to-use reagents provided with the kit. During the final step, hydrolysis of the 4-methylumbelliferyl phosphate (4-MUP) substrate via alkaline phosphatase generated fluorescent 4-methylumbelliferone (4-MU). Fluorescence intensity, measured at assay-specific excitation and emission wavelengths, was directly proportional to the TnI concentration. Quantification was achieved using the instrument’s internal calibration curve, and the final results were expressed in µg/L.

#### 2.6.4. Determination of Blood Lactate Levels

Tail-vein blood was collected using lithium-heparin-pretreated syringes to prevent clot formation and reduce glycolysis-related interference. Quantification of lactate was performed using the ABL800 FLEX blood gas analyzer produced by Radiometer^®^ (Copenhagen, Denmark). The measurement is based on an optical fluorescence electrode system, wherein lactate undergoes enzymatic oxidation by lactate oxidase, producing hydrogen peroxide (H_2_O_2_) as the reaction product (lactate + O_2_ → pyruvate + H_2_O_2_). H_2_O_2_ produced by the oxidation of lactate is detected at the sensor’s surface, where the generated electrochemical signal exhibits a stoichiometric relationship with the lactate level. All measurements were conducted promptly after sample collection to limit pre-analytical fluctuations and maintain high analytical precision.

#### 2.6.5. Determination of Serum LDH Activity

Serum lactate dehydrogenase (LDH, P–L) activity was measured using a UV–kinetic spectrophotometric assay using the AU5800 automated analyzer system (Beckman Coulter Inc.^®^, Brea, CA, USA, 2023). The assay was carried out in accordance with the methodological standards established by the International Federation of Clinical Chemistry and Laboratory Medicine (IFCC). In this enzymatic process, LDH transfers electrons from reduced nicotinamide adenine dinucleotide (NADH) to pyruvate, producing L-lactate and oxidizing NADH to NAD^+^ (pyruvate + NADH + H^+^ → L-lactate + NAD^+^). The oxidation kinetics of NADH maintain a strict stoichiometric relationship with LDH activity, enabling its catalytic rate to be accurately assessed. The decline in absorbance associated with NADH depletion was recorded continuously at 340 nm (Table S1).

### 2.7. Histopathological Procedures

Cardiac tissue samples were promptly fixed in 10% neutral buffered formalin to ensure the optimal preservation of morphological and cellular architecture. The tissues were transferred into processing cassettes and subsequently washed under a continuous stream of tap water for 24 h to eliminate excess fixative. Dehydration was carried out through a stepwise ethanol series (70%, 80%, 90%, and 100%) to remove water from the tissue specimens. The dehydrated tissues were subsequently cleared in xylol and infiltrated with molten paraffin to facilitate embedding. Paraffin blocks were sectioned at a thickness of 4–5 μm using a rotary microtome, and the resulting sections were mounted onto glass slides. For histological evaluation, serial sections obtained from the cardiac tissues of six animals per group (*n* = 6) were stained with hematoxylin and eosin (H&E) to assess overall tissue morphology. One central microscopic field from each section was examined at ×20 magnification, providing six representative images for each experimental group. Microscopic evaluations were conducted using a DP2-SAL imaging system (Olympus^®^ Inc., Tokyo, Japan), and photomicrographs were obtained for documentation purposes. Histopathological changes in the cardiac specimens were characterized by degenerative–necrotic lesions accompanied by varying levels of inflammatory cell infiltration. Histopathological injury was evaluated semi-quantitatively using a four-grade scoring scheme applied to six randomly selected microscopic fields. A score of 0 indicated the absence of detectable lesions; a score of 1 reflected mild histopathological alterations; a score of 2 corresponded to moderate tissue injury; a score of 3 denoted severe pathological changes (Table 1). All evaluations were conducted by an experienced pathologist who was blinded to the experimental groups.

### 2.8. Statistical Analyses

All statistical analyses for the biochemical and histopathological data were conducted using IBM SPSS^®^ Statistics for Windows, version 27.0 (IBM Corp., Armonk, NY, USA). Graphical data visualization was performed using GraphPad Prism^®^ version 8.0.1 (GraphPad Software, San Diego, CA, USA). All biochemical measurements are reported as mean ± standard deviation (SD). Normality of the data was evaluated using the Shapiro–Wilk test, and homogeneity of variances was determined using Levene’s test (Tables S2 and S3). When both assumptions were satisfied, group differences were analyzed using one-way analysis of variance (ANOVA), and pairwise comparisons were performed with Tukey’s Honestly Significant Difference (HSD) post hoc test. In the absence of variance homogeneity, Welch’s ANOVA was utilized for group comparisons, and the Games–Howell test was applied for post hoc multiple testing (Table S4). Histopathological scores are reported as median values along with their minimum and maximum ranges. Groupwise differences were analyzed using the nonparametric Mann–Whitney U test, with all comparisons conducted as two-tailed. A *p*-value of <0.05 was accepted as the threshold for statistical significance.

## 3. Results

### 3.1. Biochemical Findings

#### 3.1.1. Analysis of MDA and tGSH Levels in Cardiac Tissue

**Figure 1:**
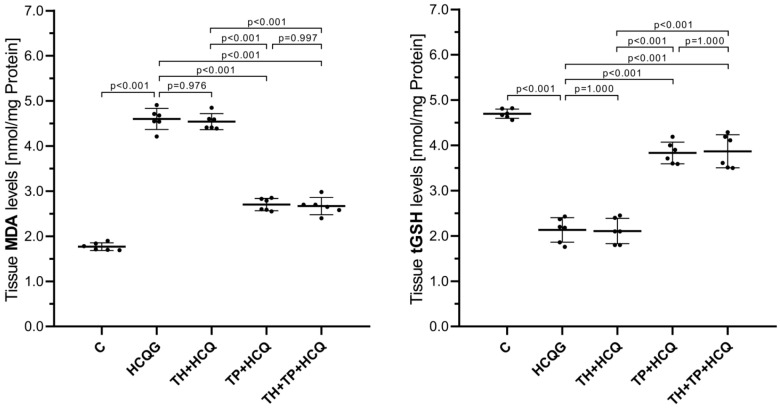
Impact of thiamine, thiamine pyrophosphate, and hydroxychloroquine administration on MDA and tGSH levels in rat cardiac tissue. All data are presented as mean ± SD (standard deviation). In the statistical evaluation, MDA was analyzed using one-way ANOVA followed by Tukey’s Honestly Significant Difference (HSD) post hoc test because the assumption of variance homogeneity was satisfied, whereas tGSH was analyzed using Welch’s ANOVA with Games–Howell post hoc comparisons due to the violation of this assumption. A significance threshold of *p* < 0.001 was adopted for all statistical analyses. For all groups, *n* = 6. **Abbreviations:** C, Healthy group; HCQG, hydroxychloroquine-only group; TH + HCQ, thiamine + HCQ group; TP + HCQ, thiamine pyrophosphate + HCQ group; TH + TP + HCQ, thiamine + thiamine pyrophosphate + HCQ group; HCQ, hydroxychloroquine; MDA, malondialdehyde; tGSH, total glutathione.

Figure 1 illustrates that hydroxychloroquine exposure markedly disrupted the redox homeostasis within cardiac tissue. Animals treated with HCQ alone (HCQG; MDA: 4.60 ± 0.23, tGSH: 2.13 ± 0.27) showed a pronounced increase in lipid peroxidation accompanied by a substantial depletion of glutathione relative to the healthy controls (C; MDA: 1.77 ± 0.09, tGSH: 4.70 ± 0.10; HCQG vs. C: MDA, *p* < 0.001; tGSH, *p* < 0.001). The co-administration of thiamine failed to exert a measurable protective effect, as the TH + HCQ group (MDA: 4.54 ± 0.18, tGSH: 2.11 ± 0.28) remained statistically indistinguishable from HCQG (MDA, *p* = 0.976; tGSH, *p* = 1.000). In contrast, thiamine pyrophosphate supplementation (TP + HCQ; MDA: 2.70 ± 0.14, tGSH: 3.83 ± 0.24) and the combined thiamine–TPP regimen (TH + TP + HCQ; MDA: 2.67 ± 0.19, tGSH: 3.87 ± 0.37) effectively suppressed the oxidative alterations induced by HCQ. Both interventions significantly inhibited the HCQ-induced rise in MDA levels (*p* < 0.001 for each vs. HCQG) while markedly suppressing the reduction in tGSH levels (*p* < 0.001 for both), demonstrating that TPP—alone or together with thiamine—effectively counteracted the pro-oxidant effects of HCQ in cardiac tissue.

#### 3.1.2. Analysis of SOD and CAT Activities in Cardiac Tissue

**Figure 2:**
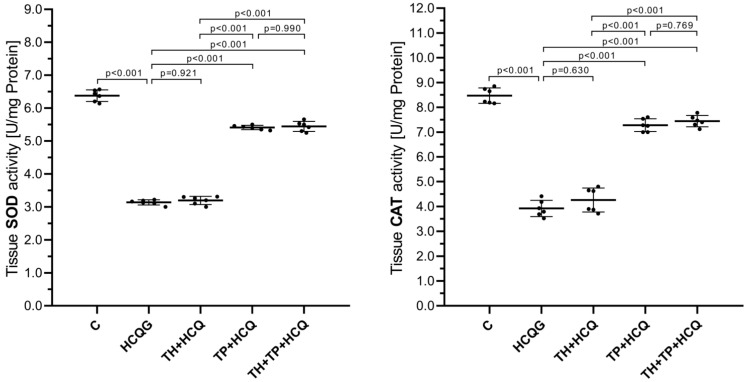
Alterations in SOD and CAT enzymatic activities in rat cardiac tissue after treatment with thiamine, thiamine pyrophosphate, and hydroxychloroquine. All data are presented as mean ± SD (standard deviation). In the statistical evaluation, SOD was analyzed using one-way ANOVA followed by Tukey’s Honestly Significant Difference (HSD) post hoc test because the assumption of variance homogeneity was satisfied, whereas CAT was analyzed using Welch’s ANOVA with Games–Howell post hoc comparisons due to the violation of this assumption. A significance threshold of *p* < 0.001 was adopted for all statistical analyses. For all groups, *n* = 6. **Abbreviations:** C, Healthy group; HCQG, hydroxychloroquine-only group; TH + HCQ, thiamine + HCQ group; TP + HCQ, thiamine pyrophosphate + HCQ group; TH + TP + HCQ, thiamine + thiamine pyrophosphate + HCQ group; HCQ, hydroxychloroquine; SOD, superoxide dismutase; CAT, catalase.

As demonstrated in Figure 2, HCQ markedly altered antioxidant enzyme activity in cardiac tissue. Animals treated with HCQ alone (HCQG; SOD: 3.14 ± 0.08, CAT: 3.92 ± 0.33) exhibited pronounced reductions in both SOD and CAT activities compared with the healthy controls (C; SOD: 6.38 ± 0.18, CAT: 8.47 ± 0.31). The differences between HCQG and C were statistically significant for both parameters (HCQG vs. C: SOD, *p* < 0.001; CAT, *p* < 0.001). The co-administration of thiamine did not modify these changes, as the TH + HCQ group (SOD: 3.20 ± 0.12, CAT: 4.26 ± 0.48) remained comparable to HCQG (TH + HCQ vs. HCQG: SOD, *p* = 0.921; CAT, *p* = 0.630). Animals receiving TPP showed a clear improvement in antioxidant enzyme activity. In the TP + HCQ group (SOD: 5.41 ± 0.07, CAT: 7.28 ± 0.26), both SOD and CAT values were markedly higher than those in HCQG (TP + HCQ vs. HCQG: SOD, *p* < 0.001; CAT, *p* < 0.001). Similarly, animals treated with the combined thiamine and TPP regimen (TH + TP + HCQ; SOD: 5.44 ± 0.15, CAT: 7.44 ± 0.23) exhibited significantly elevated enzyme activities relative to HCQG (TH + TP + HCQ vs. HCQG: SOD, *p* < 0.001; CAT, *p* < 0.001). These findings demonstrate that TPP, either alone or together with thiamine, effectively suppressed the HCQ-induced decline in SOD and CAT activity within cardiac tissue.

#### 3.1.3. Analysis of Serum TnI Levels

**Figure 3:**
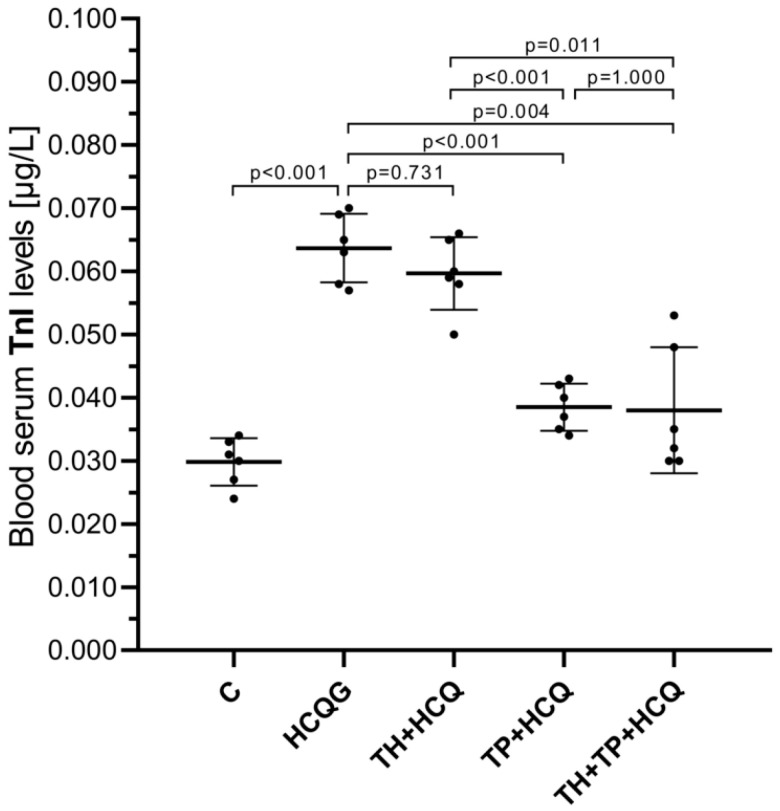
Changes in plasma TnI levels following the administration of thiamine, thiamine pyrophosphate, and hydroxychloroquine. All data are presented as mean ± SD (standard deviation). As the assumption of variance homogeneity was not satisfied, Welch’s ANOVA was employed, and intergroup differences were subsequently assessed using the Games–Howell post hoc test. A significance threshold of *p* < 0.001 was adopted for statistical analyses. For all groups, *n* = 6. **Abbreviations:** C, Healthy group; HCQG, hydroxychloroquine-only group; TH + HCQ, thiamine + HCQ group; TP + HCQ, thiamine pyrophosphate + HCQ group; TH + TP + HCQ, thiamine + thiamine pyrophosphate + HCQ group; HCQ, hydroxychloroquine; TnI, troponin I.

As shown in Figure 3, serum TnI levels were markedly elevated in animals receiving hydroxychloroquine alone (HCQG, 0.064 ± 0.01) compared with the healthy controls (C, 0.030 ± 0.00; HCQG vs. C, *p* < 0.001). The co-administration of thiamine did not modify this increase, as the TH + HCQ group (0.060 ± 0.01) exhibited TnI levels similar to those of HCQG (TH + HCQ vs. HCQG, *p* = 0.731). In contrast, animals treated with thiamine pyrophosphate (TP + HCQ, 0.039 ± 0.00) or the combined thiamine and TPP regimen (TH + TP + HCQ, 0.038 ± 0.01) displayed significantly lower serum TnI levels than both HCQG (TP + HCQ vs. HCQG, *p* < 0.001; TH + TP + HCQ vs. HCQG, *p* = 0.004) and TH + HCQ (TP + HCQ vs. TH + HCQ, *p* < 0.001; TH + TP + HCQ vs. TH + HCQ, *p* = 0.011). These findings indicate that TPP effectively inhibited the HCQ-induced rise in serum TnI levels.

#### 3.1.4. Analysis of Blood Lactate Levels

**Figure 4:**
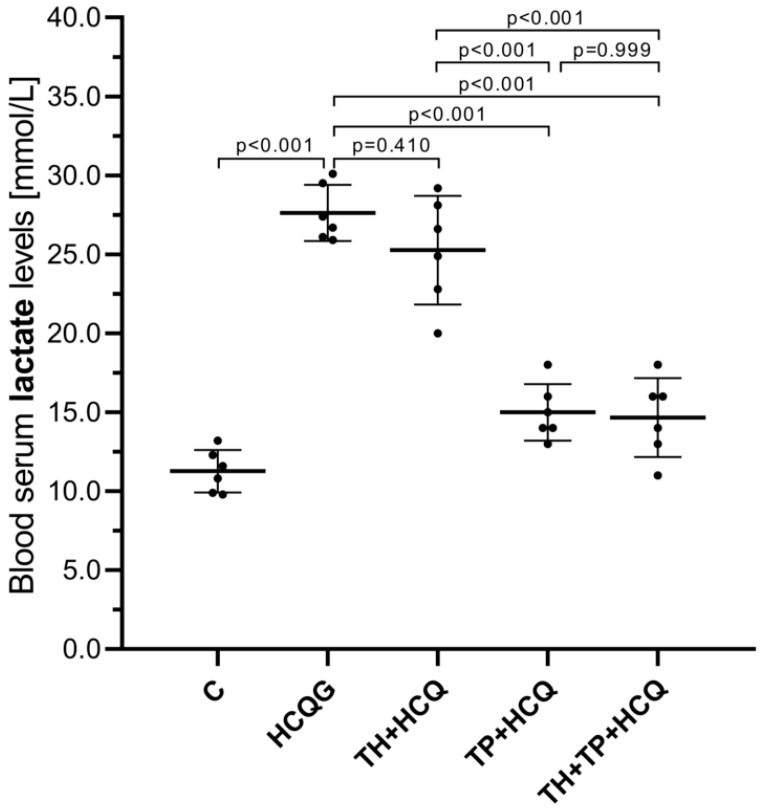
Alterations in blood serum lactate levels in response to treatment with thiamine, thiamine pyrophosphate, and hydroxychloroquine. All data are presented as mean ± SD (standard deviation). Statistical analyses were performed using one-way ANOVA, and given that the assumption of variance homogeneity was satisfied, post hoc multiple comparisons were conducted using Tukey’s Honestly Significant Difference (HSD) test. A significance threshold of *p* < 0.001 was adopted for statistical analyses. For all groups, *n* = 6. **Abbreviations:** C, Healthy group; HCQG, hydroxychloroquine-only group; TH + HCQ, thiamine + HCQ group; TP + HCQ, thiamine pyrophosphate + HCQ group; TH + TP + HCQ, thiamine + thiamine pyrophosphate + HCQ group; HCQ, hydroxychloroquine.

As shown in Figure 4, blood lactate levels were markedly increased in animals receiving HCQ alone (HCQG, 27.62 ± 1.78) compared with the healthy controls (C, 11.27 ± 1.35). Thiamine co-administration did not meaningfully alter this response, as lactate values in the TH + HCQ group (25.27 ± 3.44) remained statistically comparable to those of HCQG (*p* = 0.410). In contrast, the introduction of TPP produced a clear corrective effect, with substantially reduced lactate levels observed in both the TP + HCQ (15.00 ± 1.79) and TH + TP + HCQ groups (14.67 ± 2.50). These reductions were significant when compared with HCQG (TP + HCQ vs. HCQG, *p* < 0.001; TH + TP + HCQ vs. HCQG, *p* < 0.001) and TH + HCQ (TP + HCQ vs. TH + HCQ, *p* < 0.001; TH + TP + HCQ vs. TH + HCQ, *p* < 0.001). Collectively, these findings demonstrate that TPP effectively counterbalanced the HCQ-induced elevation in blood lactate, thereby mitigating HCQ-driven metabolic disturbance.

#### 3.1.5. Analysis of Serum LDH Activity

**Figure 5:**
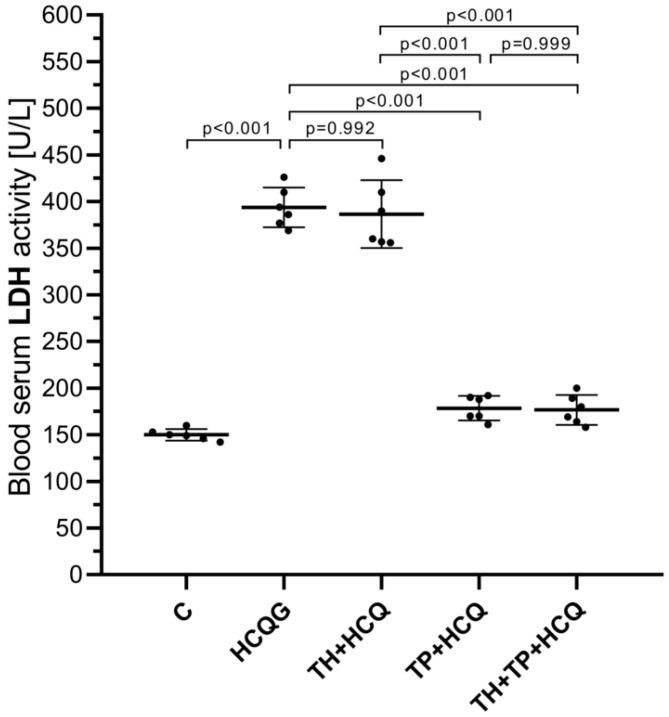
Changes in blood serum LDH activity following administration of thiamine, thiamine pyrophosphate, and hydroxychloroquine. All data are presented as mean ± SD (standard deviation). As the assumption of variance homogeneity was not satisfied, Welch’s ANOVA was employed, and intergroup differences were subsequently assessed using the Games–Howell post hoc test. A significance threshold of *p* < 0.001 was adopted for statistical analyses. For all groups, *n* = 6. **Abbreviations:** C, Healthy group; HCQG, hydroxychloroquine-only group; TH + HCQ, thiamine + HCQ group; TP + HCQ, thiamine pyrophosphate + HCQ group; TH + TP + HCQ, thiamine + thiamine pyrophosphate + HCQ group; HCQ, hydroxychloroquine; LDH, lactate dehydrogenase.

Figure 5 shows that exposure to HCQ resulted in a marked elevation in serum LDH activity, with the HCQG group (393.67 ± 21.25) displaying substantially higher levels than the healthy controls (C, 150.00 ± 6.16). The LDH values recorded in the TH + HCQ group (386.50 ± 36.35) were statistically indistinguishable from those of HCQG (*p* = 0.992), indicating that thiamine alone did not meaningfully modify this response. In contrast, the administration of TPP produced a clear inhibitory effect on LDH elevation, as evidenced by the markedly reduced levels measured in the TP + HCQ (178.50 ± 13.08) and TH + TP + HCQ groups (176.67 ± 15.97). These reductions were significant when compared with both HCQG (TP + HCQ vs. HCQG, *p* < 0.001; TH + TP + HCQ vs. HCQG, *p* < 0.001) and TH + HCQ (TP + HCQ vs. TH + HCQ, *p* < 0.001; TH + TP + HCQ vs. TH + HCQ, *p* < 0.001). Collectively, these findings indicate that TPP effectively counteracted the HCQ-driven increase in serum LDH activity.

### 3.2. Histopathological Findings

**Figure 6:**
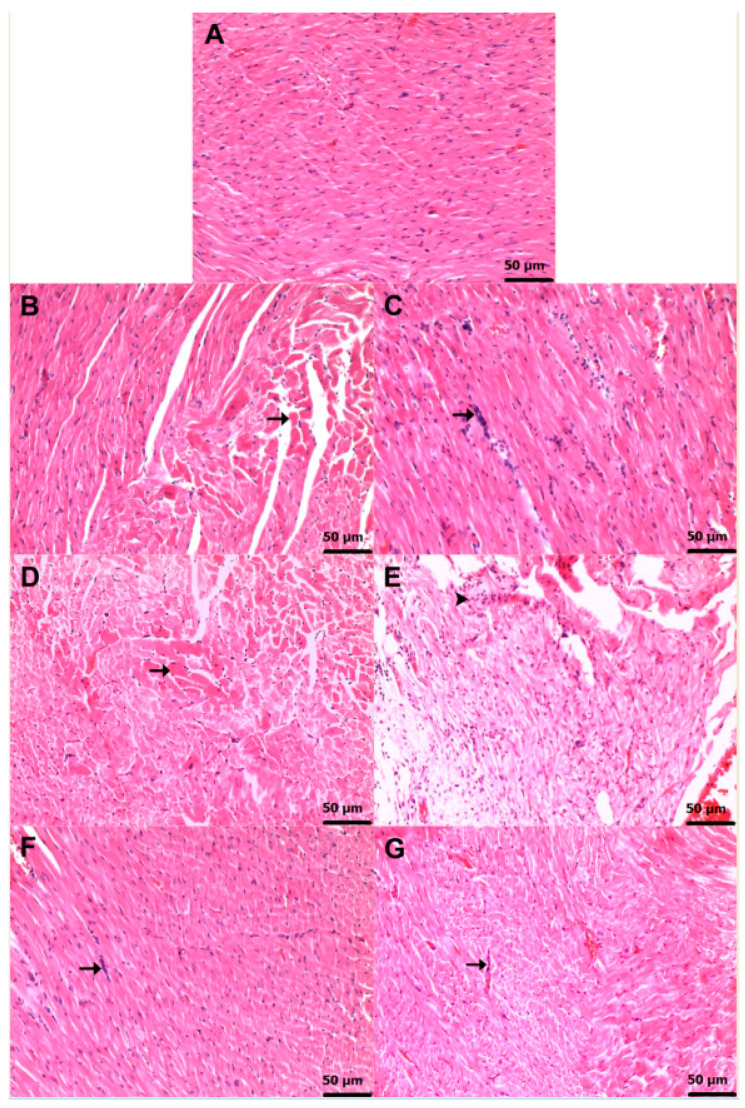
Histopathological features of cardiac tissue in experimental groups. Representative photomicrographs of cardiac tissue from each experimental group are shown. (**A**) C group demonstrating preserved myocardial architecture with intact myofibers and normal interstitial spaces (H&E, ×20). (**B**) HCQG group exhibiting severe myocyte degeneration (**→**) (H&E, ×20). (**C**) HCQG group displaying moderate mononuclear inflammatory infiltration within the interstitial areas (**→**) (H&E, ×20). (**D**) TH + HCQ group showing persistent severe myocyte degeneration (**→**) comparable to HCQG (H&E, ×20). (**E**) TH + HCQ group with moderate mononuclear cell infiltration in the interstitium (arrowhead) (H&E, ×20). (**F**) TP + HCQ group demonstrating mild mononuclear inflammatory infiltration (**→**) with partially preserved myocardial morphology (H&E, ×20). (**G**) TH + TP + HCQ group showing mild mononuclear cell infiltration (**→**) and generally preserved myocardial architecture (H&E, ×20). **Abbreviations:** C, Healthy group; HCQG, HCQ-only group; TH + HCQ, thiamine + HCQ group; TP + HCQ, thiamine pyrophosphate + HCQ group; TH + TP + HCQ, thiamine + thiamine pyrophosphate + HCQ group; HCQ, hydroxychloroquine.

Figure 6A illustrates the expected histoarchitectural organization of the myocardium in the healthy group, characterized by intact myofibers and unremarkable interstitial spaces. In contrast, HCQ administration resulted in a marked deterioration of myocardial morphology. Animals in the HCQG cohort exhibited severe (grade 3) myocyte degeneration (Figure 6B), accompanied by a moderate (grade 2) mononuclear inflammatory infiltrate within the interstitium (Figure 6C). Thiamine did not mitigate these HCQ-induced alterations, as the TH + HCQ group retained both severe (grade 3) myocyte injury and moderate (grade 2) inflammatory infiltration (Figure 6D,E). Notably, treatment with TPP produced a clearly attenuated histopathological profile. Both the TP + HCQ (Figure 6F) and TH + TP + HCQ (Figure 6G) groups only displayed mild (grade 1) mononuclear cell infiltration and showed no evidence of severe (grade 3) myocyte degeneration, with myocardial architecture largely preserved. A detailed semi-quantitative assessment of the cardiac histopathological findings, together with the corresponding intergroup statistical evaluations and *p*-values, is provided in Table 2.

## 4. Discussion

**Figure 7:**
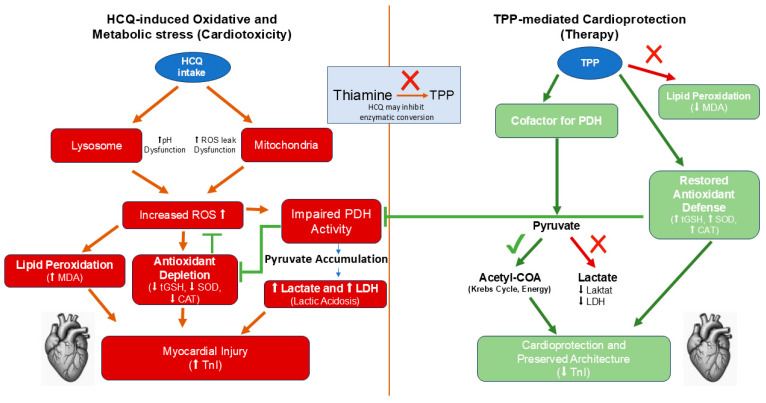
Schematic overview of HCQ-induced oxidative stress and the cardioprotective mechanisms of TPP. **Abbreviations:** HCQ, Hydroxychloroquine; TPP, thiamine pyrophosphate; ROS, reactive oxygen species; PDH, pyruvate dehydrogenase; MDA, malondialdehyde; tGSH, total glutathione; SOD, superoxide dismutase; CAT, catalase; TnI, troponin I; LDH, lactate dehydrogenase.

In our study, the protective effects of thiamine, TPP, and TTPC against HCQ-induced cardiomyopathy were examined through biochemical and histopathological analyses and were comparatively evaluated. In the animals to which HCQ was administered, MDA levels in cardiac tissue—as well as serum TnI, serum LDH, and blood lactate levels—were found to be markedly elevated, whereas cardiac tissue levels of tGSH, SOD, and CAT were observed to be significantly diminished. The findings of our study provide compelling evidence that HCQ precipitates significant oxidative damage. To improve clarity regarding the mechanistic interplay between HCQ-induced oxidative stress and the cardioprotective actions of TPP, a schematic overview of the pathways is provided in Figure 7. The excessive generation of reactive oxygen species (ROS), which play essential roles in numerous physiological functions and in the maintenance of cellular homeostasis, can result in the development of oxidative stress [31]. Following HCQ administration in animals, the increased MDA identified in cardiac tissue constitutes the terminal product of lipid peroxidation (LPO) and is widely recognized as a pivotal biomarker indicative of oxidative injury [32]. Previous studies have also proposed that HCQ may induce oxidative stress and cardiotoxicity by enhancing ROS generation within mitochondrial respiratory chains and lysosomal compartments [33]. It is well established that mitochondrial dysfunction precipitates energy depletion, oxidative stress, and inflammatory responses within cardiac myocytes [34]. Recent research has highlighted that coordination between nuclear and mitochondrial genomes is crucial for maintaining oxidative phosphorylation and redox balance. The tissue-specific modulation of nuclear-encoded mitochondrial Complex I genes can alter ROS generation and cellular oxidative status [35]. This broader evidence supports the interpretation that HCQ-associated cardiotoxicity may involve the secondary disruption of mitochondrial regulatory pathways and oxidative phosphorylation dynamics. Onaloglu et al. emphasized that the pathogenesis of HCQ-induced oxidative stress is fundamentally linked to elevations in MDA levels, a downstream product of ROS-mediated LPO [36]. In our study, the significant elevation of MDA levels in the HCQ-treated animal group indicates that HCQ induces oxidative stress through ROS and LPO pathways, a finding that is consistent with previous reports in the literature [37].

The literature indicates that tGSH, SOD, and CAT constitute key antioxidant defense mechanisms that protect cells against oxidative stress [38]. These antioxidants are molecules that prevent or alleviate oxidative processes [39]. In the study conducted by Amer et al., the antioxidant quercetin was shown to prevent the HCQ-associated reduction in total antioxidant capacity [37]. Abdelmageed et al. reported that HCQ administration was associated with elevated MDA levels accompanied by reductions in GSH and SOD levels [40]. Topak et al. reported that CAT activity was significantly lower in HCQ-treated groups compared with the control groups [41]. Consistent with these reports, our findings demonstrate that the levels of the antioxidant defense components—tGSH, SOD, and CAT—were significantly decreased in the HCQ-treated animal group.

Serum cardiac TnI concentration serves as an early indicator of myocardial injury. An increase in serum TnI is closely associated with the severity of myocardial cell injury and is regarded as a highly selective biomarker of cardiac damage [37]. The literature indicates that prolonged HCQ use may lead to cardiomyopathy, resulting in myocardial injury and cellular death and thereby causing elevations in TnI levels [42]. Our results parallel those of previous investigations, showing that a significant increase in plasma TnI levels occurred in the HCQ-only treated animal groups, in accordance with the existing literature [43]. HCQ accumulates extensively within tissues and, over time, may elevate TnI levels, resulting in myocyte injury and ultimately contributing to the development of cardiomyopathy [44].

HCQ-induced cardiac muscle injury was evaluated through the measurement of LDH enzyme levels. In cases of cardiomyopathy, alterations in LDH enzymatic activity may occur. LDH is a bidirectional enzyme that catalyzes the interconversion of lactate and pyruvate [12]. Among the conditions that can cause elevated LDH levels in the bloodstream are acute myocardial infarction events [45]. Lactate is both produced and utilized within tissues such as the cardiac muscle. Lactate, an important biomarker reflecting disease severity, exhibits elevated blood levels in conditions such as heart failure, where oxygen availability is reduced [46]. The underlying mechanism is that LDH converts pyruvate to lactate when oxygen metabolism is disrupted [47]. Onsia et al. reported that HCQ overdose resulted in arrhythmias and an elevation in lactate levels [48]. Zhang et al. reported that patients presenting with elevated baseline serum LDH levels had an increased risk of being diagnosed with heart failure [18]. Biguetti et al. noted that LDH enzyme activity was elevated in HCQ-associated myopathy [19]. Consistent with the aforementioned studies, our findings demonstrated that blood lactate levels and serum LDH levels were significantly elevated in the group receiving HCQ.

As previously mentioned, thiamine is also known as vitamin B1 [20]. Thiamine deficiency is known to result in the development of lactic acidosis [49]. Studies investigating the protective effects of thiamine on cardiac tissue are available in the literature [50]. Rankovic et al. reported that thiamine prevented the increase in cardiac MDA levels and the concomitant reductions in tGSH, SOD, and CAT levels [51]. It has been noted in the literature that thiamine administration leads to an improvement in blood lactate levels toward normal values [52]. Saetang et al. demonstrated that thiamine administration prevented elevations in cardiac biomarker levels, such as TnI and LDH [53]. However, in contrast to these reports, no significant alterations were observed in oxidative stress parameters or cardiac biomarkers in the group to which we administered thiamine despite HCQ-induced oxidative stress. Coskun et al. stated that TPP exhibited a protective effect against drug-induced cardiotoxicity through oxidative agents, whereas thiamine was insufficient in preventing this oxidative damage [54]. Our experimental results indicate that thiamine possesses only limited protective efficacy and is insufficient in the control of HCQ-induced mitochondrial dysfunction.

In our study, the effect of TPP against HCQ-induced cardiac injury was also investigated. As is well known, TPP is the active metabolite of thiamine [55]. TPP is a cofactor of pyruvate dehydrogenase and plays a crucial role in the conversion of pyruvate to acetyl–coenzyme A. In the absence of adequate TPP, pyruvate is converted to lactate, and the resulting accumulation of lactic acid may lead to the development of lactic acidosis [22]. Increased lactic acid production may augment oxygen consumption, while the associated reduction in myocardial energy generation can give rise to adverse cardiac outcomes [56]. It has also been noted in the literature that TPP may be utilized in the prevention of cardiac disorders [57]. Emir et al. demonstrated that TPP prevented the increase in cardiac MDA and TnI levels and the reduction in GSH levels [58]. Indeed, in our study, marked improvements in the biochemical parameters were observed in the group treated with TPP. This observation—namely that MDA, tGSH, SOD, and CAT levels in the TPP-treated group approximated those of the healthy controls—indicates its antioxidant efficacy and aligns with previous reports [59]. Isık et al., in their study examining the effects of TPP on oxidative injury and lactic acidosis, stated that lactate and LDH levels remained close to those of the healthy group [25]. Similarly, our findings provide evidence that TPP prevents elevations in blood TnI, lactate, and LDH levels. However, in the TTPC-treated group, oxidant, antioxidant, cardiac biomarker, and lactate levels were found to be similar to those in the TPP-treated group, and the differences between them were not statistically significant. This finding indicates that the protective effect against potential toxicity is primarily attributable to TPP. The absence of an additive or synergistic effect in the combined thiamine + TPP group compared with TPP alone may be explained by metabolic and enzymatic constraints. Since TPP is the biologically active form of thiamine and directly functions as a cofactor for key mitochondrial enzymes involved in energy metabolism, sufficient intracellular TPP availability may represent a limiting factor. Under conditions in which the enzymatic conversion of thiamine to TPP may be impaired or enzymatically saturated, additional thiamine supplementation is unlikely to confer further benefit. Accordingly, the cardioprotective effects observed in the present study appear to be primarily mediated by TPP itself, with no additional advantage provided by combined thiamine administration. Similar findings have been reported in the literature [60].

Our histopathological findings are consistent with and corroborate our biochemical results. In the HCQ-administered group, grade 3 degeneration of cardiac myocytes and grade 2 mononuclear cell infiltrations within the interstitial spaces were observed. Our results demonstrate that, consistent with the literature, severe cardiac tissue injury occurs following HCQ treatment [37]. Similarly, Ibrahim et al. stated in their study that HCQ administration resulted in severe myocardial injury, characterized by interstitial fibrosis, myofibrillar loss, vacuolization, and inflammation [61]. No histopathological improvement was observed in the group that received both thiamine and HCQ. Thiamine was unable to prevent severe myocyte degeneration and grade 2 mononuclear cell infiltrations induced by HCQ. In contrast, only mild mononuclear cell infiltration was observed in the groups treated with TPP and TTPC. This finding demonstrates that TPP is capable of substantially preventing cardiac injury, whereas, in contrast, thiamine does not display a similar effect. The results obtained are consistent with previous studies in the literature [54,60]. These findings should be interpreted with caution due to the experimental nature of this study. It should be borne in mind that HCQ exposure may exacerbate myocardial injury in patients with myocarditis or pericarditis, which are already characterized by inflammatory and oxidative stress. Further clinical studies are required to elucidate the clinical significance of these findings and to determine the potential role of TPP in preventing HCQ-associated myocardial injury in relevant patient populations.

## 5. Conclusions

The present study provides compelling biochemical and histopathological evidence that HCQ induces a pronounced oxidative stress environment, culminating in substantial myocardial injury. Taken together, our findings show that HCQ causes cardiac tissue injury, as demonstrated by elevations in oxidative damage markers and alterations in key metabolic enzymes. TPP administration markedly ameliorated these pathological alterations, indicating a robust protective capacity against HCQ-induced myocardial dysfunction. In contrast, thiamine failed to confer a comparable benefit, and the combined administration of thiamine and TPP (TTPC) did not exhibit superiority over TPP alone. The reasons underlying the insufficient cardioprotective efficacy of thiamine remain to be fully elucidated; however, one plausible explanation is that HCQ may interfere with the enzymatic conversion of thiamine to its active cofactor, TPP. Taken together, these observations indicate that TPP supplementation may constitute a viable protective approach in settings requiring the use of HCQ, a drug associated with an increased risk of cardiomyopathy. Further mechanistic and translational investigations are warranted to validate these findings and to clarify the therapeutic potential of TPP in preventing drug-associated myocardial injury.

## 6. Limitations

This study has several noteworthy limitations that should be considered when interpreting the findings. First, our investigation was conducted exclusively in an experimental animal model; thus, the translational applicability of these results to human physiology remains uncertain, and confirmation through well-designed clinical studies is required. Second, although this study was designed to compare the protective effects of thiamine and its active metabolite TPP, neither circulating thiamine concentrations nor TPP levels were measured. Furthermore, despite the mechanistic premise that HCQ may impair the enzymatic conversion of thiamine to TPP, no assessments of thiamine pyrophosphokinase activity or expression were conducted. The absence of these biochemical measurements limits the ability to directly confirm the hypothesized disruption of thiamine–TPP metabolism. Third, HCQ and the protective agents were administered for only seven days, a duration that is substantially shorter than the month- to year-long therapeutic courses typically encountered in clinical practice. The relatively short exposure period may fail to fully capture or adequately reflect the cumulative and progressive cardiotoxic effects typically associated with prolonged HCQ administration. Fourth, this study assessed cardiac injury primarily through tissue-level biochemical analyses and histopathological evaluation, supplemented by circulating markers of cardiac and metabolic disturbance. However, no gene expression studies related to vitamin B_1_ metabolism, oxidative pathways, or mitochondrial function were conducted. Likewise, thiamine- or TPP-associated metabolic genes, transporters, and regulatory mechanisms were not investigated. Furthermore, no systematic examination of extra-cardiac organs or broader physiological parameters was performed. These limitations reduce the mechanistic depth of our study and constrain the ability to determine how cardiac alterations interface with whole-body thiamine metabolism and systemic toxicity. Fifth, only male Wistar rats were included in this study. Given the well-established sex-related differences in cardiac physiology, drug metabolism, and vulnerability to oxidative and metabolic stress, the absence of female animals limits the generalizability of the findings and restricts any conclusions regarding potential sex-specific responses to HCQ, thiamine, or TPP. Sixth, although HCQ-induced cardiotoxicity was discussed primarily in relation to mitochondrial dysfunction, no direct evaluations of mitochondrial structure or function were performed. Key parameters such as myocardial ATP content, mitochondrial membrane potential, respiratory chain complex activities, and mitochondrial-specific oxidative stress markers were not assessed. Future investigations should, therefore, incorporate dedicated mitochondrial functional assays and molecular markers of mitochondrial integrity to more clearly delineate the mechanistic pathways implicated in the present study. Seventh, a single dosing regimen was used for HCQ, thiamine, and TPP, and the high-dose, short-term HCQ exposure model may not fully reflect the chronic, low-dose use encountered in clinical practice. The absence of alternative doses, treatment schedules, and extended follow-up limits dose–response interpretation. Future studies employing lower doses and longer administration periods are therefore needed to better characterize therapeutic windows, efficacy thresholds, and safety profiles. Eighth, the potential effects of thiamine and TPP on the pharmacokinetics and tissue distribution of HCQ were not evaluated. In the absence of dedicated pharmacokinetic analyses, potential interactions influencing HCQ absorption, distribution, metabolism, or excretion cannot be excluded. Accordingly, future studies incorporating comprehensive pharmacokinetic assessments are warranted to elucidate whether thiamine or TPP modulates the tissue distribution or systemic exposure of HCQ. Finally, the absence of a direct comparison between TPP and other well-established antioxidants also represents a limitation, and expanding our study in this manner in the future would allow more definitive conclusions to be drawn regarding their cardioprotective efficacy.

## Figures and Tables

**Table 1:** Semi-quantitative four-grade histopathological scoring system.

Grade	Severity	Cardiac Myocyte Degeneration	Mononuclear Cell Infiltration
0	None	No detectable degeneration	No detectable inflammatory cells
1	Mild	≤5% of the field	≤50 inflammatory cells in the field
2	Moderate	6–14% of the field	51–99 inflammatory cells in the field
3	Severe	≥15% of the field	≥100 inflammatory cells in the field

**Table 2:** Semi-quantitative analysis of myocardial histopathological alterations in rats.

	Histopathological Grading Data	
Groups	Cardiac Myocyte Degeneration	Myocardial Interstitial Mononuclear Cell Infiltration
C	0.00 (0.00–0.00)	0.00 (0.00–0.00)
HCQG	3.00 (2.00–3.00)	2.00 (1.00–2.00)
TH + HCQ	3.00 (2.00–3.00)	2.00 (1.00–2.00)
TP + HCQ	0.00 (0.00–2.00)	1.00 (0.00–1.00)
TH + TP + HCQ	0.00 (0.00–2.00)	1.00 (0.00–1.00)
Group comparisons	*p*-values	
C vs. HCQG	0.001	0.001
C vs. TH + HCQ	0.001	0.001
C vs. TP + HCQ	0.140	0.005
C vs. TH + TP + HCQ	0.140	0.005
HCQG vs. TH + HCQ	1.000	1.000
HCQG vs. TP + HCQ	0.003	0.006
HCQG vs. TH + TP + HCQ	0.003	0.006
TH + HCQ vs. TP + HCQ	0.003	0.006
TH + HCQ vs. TH + TP + HCQ	0.003	0.006
TP + HCQ vs. TH + TP + HCQ	1.000	1.000

Data are presented as median values with corresponding minimum–maximum ranges. Statistical analyses were performed using the Mann–Whitney U test. All groups consisted of six samples (*n* = 6). **Abbreviations:** C, healthy group; HCQG, hydroxychloroquine-only group; TH + HCQ, thiamine + HCQ group; TP + HCQ, thiamine pyrophosphate + HCQ group; TH + TP + HCQ, thiamine + thiamine pyrophosphate + HCQ group; HCQ, hydroxychloroquine.

## Data Availability

The original contributions presented in this study are included in this manuscript/Supplementary Materials. Further inquiries can be directed to the corresponding author.

## Supplementary Materials

The following supporting information can be downloaded at: https://www.mdpi.com/article/10.3390/life16010037/s1, Table S1. Kinetic measurement of LDH activity; Table S2. Evaluation of distributional normality of biochemical parameters in rat heart tissue and blood using the Shapiro–Wilk test; Table S3. Levene’s test results for the homogeneity of variances assumption for biochemical variables; Table S4. Comparison of *p*-values for the effects of thiamine, thiamine pyrophosphate, and hydroxychloroquine on biochemical parameters in rat heart tissue and blood.
