# Supplementary material for: Comparative Study on the Protective Effect of Thiamine and Thiamine Pyrophosphate Against Hydroxychloroquine-Induced Cardiomyopathy in Rats
Source: Life (Basel). 2025 Dec 25;16(1):37. doi: 10.3390/life16010037 (PMC12843037; doi:10.3390/life16010037)
Supplement: Supplementary file 1 [file life-16-00037-s001.zip › Table S4-R2.pdf]

Table S4. Comparison of p-values for the effects of thiamine, thiamine pyrophosphate, and hydroxychloroquine on biochemical parameters in rat heart tissue and blood

	Posthoc test p-values						
**Group comparisons**	**MDA***	**tGSH****	**SOD***	**CAT****	**TnI****	**Lactate***	**LDH****
**C vs. HCQG**	<0.001	<0.001	<0.001	<0.001	<0.001	<0.001	<0.001
**C vs. TH+HCQ**	<0.001	<0.001	<0.001	<0.001	<0.001	<0.001	<0.001
**C vs. TP+HCQ**	<0.001	<0.001	<0.001	<0.001	0.017	0.064	0.011
**C vs. TH+TP+HCQ**	<0.001	0.011	<0.001	<0.001	0.412	0.108	0.042
**HCQG vs. TH+HCQ**	0.976	1.000	0.921	0.630	0.731	0.410	0.992
**HCQG vs. TP+HCQ**	<0.001	<0.001	<0.001	<0.001	<0.001	<0.001	<0.001
**HCQG vs. TH+TP+HCQ**	<0.001	<0.001	<0.001	<0.001	0.004	<0.001	<0.001
**TH+HCQ vs. TP+HCQ**	<0.001	<0.001	<0.001	<0.001	<0.001	<0.001	<0.001
**TH+HCQ vs. TH+TP+HCQ**	<0.001	<0.001	<0.001	<0.001	0.011	<0.001	<0.001
**TP+HCQ vs. TH+TP+HCQ**	0.997	1.000	0.990	0.769	1.000	0.999	0.999
**F value**	320.514	113.261[a]	805.377	227.893[a]	34.719[a]	59.234	199.517[a]
**df (df1 / df2)**	4 / 25	4 / 11.566	4 / 25	4 / 12.352	4 / 12.261	4 / 25	4 / 11.461
**p**	<0.001	<0.001[b]	<0.001	<0.001[b]	<0.001[b]	<0.001	<0.001[b]

Footnotes: *All statistical analyses were performed using one-way ANOVA, followed by Tukey's honestly significant difference (HSD) test for post hoc multiple comparisons when the assumption of homogeneity of variances was met. **Welch's ANOVA was applied when this assumption was violated, and post hoc comparisons were conducted using the Games–Howell test. [a] indicates values that are asymptotically F-distributed; [b] denotes p-values derived from Welch's ANOVA.

Abbreviations: C, healthy group; HCQG, hydroxychloroquine-only group; TH + HCQ, thiamine + HCQ group; TP + HCQ, thiamine pyrophosphate + HCQ group; TH + TP +HCQ, thiamine + thiamine pyrophosphate + HCQ group; HCQ, hydroxychloroquine; MDA, malondialdehyde; tGSH, total glutathione; SOD, superoxide dismutase; CAT, catalase; TnI, troponin I; LDH, lactate dehydrogenase; df, degrees of freedom.
