# Supplementary material for: Comparative Study on the Protective Effect of Thiamine and Thiamine Pyrophosphate Against Hydroxychloroquine-Induced Cardiomyopathy in Rats
Source: Life (Basel). 2025 Dec 25;16(1):37. doi: 10.3390/life16010037 (PMC12843037; doi:10.3390/life16010037)
Supplement: Supplementary file 1 [file life-16-00037-s001.zip › Table S2-R2.pdf]

Table S2. Evaluation of distributional normality of biochemical parameters in rat heart tissue and blood using the Shapiro–Wilk test

			Biochemical Variables						
		**Shapiro -Wilk**	**MDA**	**tGSH**	**SOD**	**CAT**	**TnI**	**Lactate**	**LDH**
**Groups**	**C**	**statistic**	0.885	0.936	0.920	0.837	0.950	0.938	0.977
		**df**	6	6	6	6	6	6	6
		**sig.**	0.295	0.626	0.508	0.124	0.739	0.644	0.937
	**HCQG**	**statistic**	0.951	0.909	0.932	0.970	0.917	0.872	0.963
		**df**	6	6	6	6	6	6	6
		**sig.**	0.746	0.428	0.599	0.893	0.483	0.233	0.841
	**TH+HCQ**	**statistic**	0.841	0.877	0.900	0.835	0.921	0.963	0.860
		**df**	6	6	6	6	6	6	6
		**sig.**	0.132	0.255	0.374	0.119	0.514	0.844	0.190
	**TP+HCQ**	**statistic**	0.833	0.919	0.977	0.890	0.925	0.933	0.861
		**df**	6	6	6	6	6	6	6
		**sig.**	0.115	0.501	0.936	0.318	0.540	0.607	0.191
	**TH+TP+ HCQ**	**statistic**	0.942	0.823	0.968	0.998	0.812	0.972	0.960
		**df**	6	6	6	6	6	6	6
		**sig.**	0.678	0.094	0.877	1.000	0.075	0.907	0.821

Footnotes: The datasets for MDA, tGSH, SOD, CAT, TnI, lactate, and LDH satisfied the normality criterion; therefore, group comparisons were conducted using analysis of variance (ANOVA).

Abbreviations: C, healthy group; HCQG, hydroxychloroquine-only group; TH + HCQ, thiamine + HCQ group; TP + HCQ, thiamine pyrophosphate + HCQ group; TH + TP + HCQ, thiamine + thiamine pyrophosphate + HCQ group; HCQ, hydroxychloroquine; MDA, malondialdehyde; tGSH, total glutathione; SOD, superoxide dismutase; CAT, catalase; TnI, troponin I; LDH, lactate dehydrogenase; df, degrees of freedom; sig, significance.
