# Supplementary material for: Comparative Study on the Protective Effect of Thiamine and Thiamine Pyrophosphate Against Hydroxychloroquine-Induced Cardiomyopathy in Rats
Source: Life (Basel). 2025 Dec 25;16(1):37. doi: 10.3390/life16010037 (PMC12843037; doi:10.3390/life16010037)
Supplement: Supplementary file 1 [file life-16-00037-s001.zip › Table S3-R2.pdf]

Table S3. Levene's test results for the homogeneity of variances assumption for biochemical variables

	Biochemical Variables						
	**MDA**	**tGSH**	**SOD**	**CAT**	**TnI**	**Lactate**	**LDH**
**Levene's statistic**	0.754	3.909	2.146	3.928	3.366	1.856	4.525
**df1**	4	4	4	4	4	4	4
**df2**	25	25	25	25	25	25	25
**sig.**	0.565	0.013	0.105	0.013	0.025	0.150	0.007

Footnotes: Since the assumption of homogeneity of variances was met, Tukey's honestly significant difference (HSD) test was employed for post hoc comparisons of MDA, SOD, and lactate. For tGSH, CAT, TnI, and LDH, the assumption was violated; therefore, the Games–Howell test was applied.
Abbreviations: MDA, malondialdehyde; tGSH, total glutathione; SOD, superoxide dismutase; CAT, catalase; TnI, troponin I; LDH, lactate dehydrogenase; df, degrees of freedom; sig, significance.
