# Supplementary figures and images for: Comparative Study on the Protective Effect of Thiamine and Thiamine Pyrophosphate Against Hydroxychloroquine-Induced Cardiomyopathy in Rats
Source: Life (Basel). 2025 Dec 25;16(1):37. doi: 10.3390/life16010037 (PMC12843037; doi:10.3390/life16010037)

**Table S1.** Kinetic measurement of LDH activity

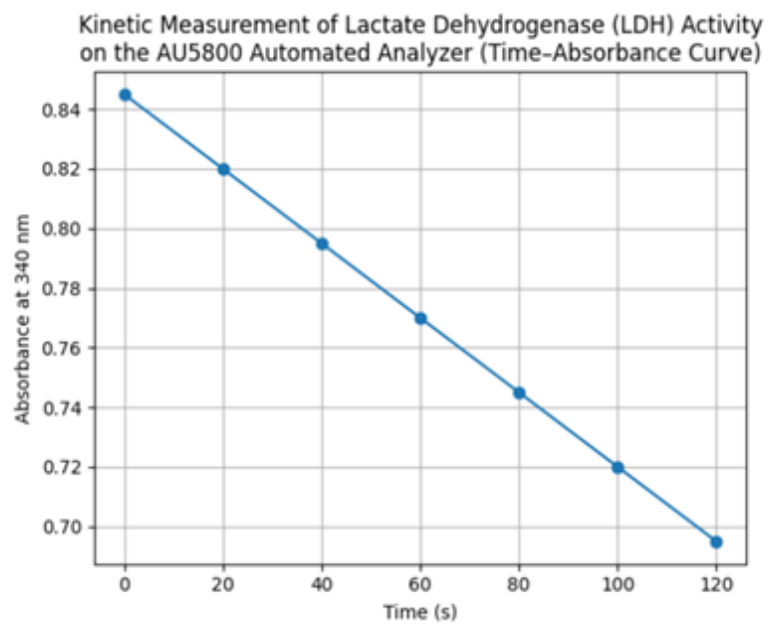

Supplement: Supplementary file 1 [file life-16-00037-s001.zip › Table S1-R2.pdf]
